# Deep Learning-Based Post-Processing of Real-Time MRI to Assess and Quantify Dynamic Wrist Movement in Health and Disease

**DOI:** 10.3390/diagnostics11061077

**Published:** 2021-06-11

**Authors:** Karl Ludger Radke, Lena Marie Wollschläger, Sven Nebelung, Daniel Benjamin Abrar, Christoph Schleich, Matthias Boschheidgen, Miriam Frenken, Justus Schock, Dirk Klee, Jens Frahm, Gerald Antoch, Simon Thelen, Hans-Jörg Wittsack, Anja Müller-Lutz

**Affiliations:** 1 Department of Diagnostic and Interventional Radiology, Medical Faculty, University Dusseldorf, D-40225 Dusseldorf, Germany; Ludger.Radke@med.uni-duesseldorf.de (K.L.R.); Sven.Nebelung@med.uni-duesseldorf.de (S.N.); Danielbenjamin.Abrar@med.uni-duesseldorf.de (D.B.A.); Christoph.Schleich@radiologie-duesseldorf-mitte.de (C.S.); Matthias.Boschheidgen@med.uni-duesseldorf.de (M.B.); Miriam.Frenken@med.uni-duesseldorf.de (M.F.); Justus.Schock@med.uni-duesseldorf.de (J.S.); Dirk.Klee@med.uni-duesseldorf.de (D.K.); Antoch@med.uni-duesseldorf.de (G.A.); Hans-Joerg.Wittsack@med.uni-duesseldorf.de (H.-J.W.); Anja.Lutz@med.uni-duesseldorf.de (A.M.-L.); 2 Department of Orthopaedics and Trauma Surgery, Medical Faculty, University Dusseldorf, D-40225 Dusseldorf, Germany; Simon.Thelen@med.uni-duesseldorf.de; 3 Department of General Pediatrics, University Children’s Hospital, Heinrich-Heine-University Dusseldorf, D-40225 Dusseldorf, Germany; 4 Biomedizinische NMR, Max-Planck Institute for Biophysical Chemistry, D-37077 Goettingen, Germany; jfrahm@gwdg.de

**Keywords:** magnetic resonance imaging, scapholunate ligament injury, carpal instability, real-time, deep learning, dynamic instability

## Abstract

While morphologic magnetic resonance imaging (MRI) is the imaging modality of choice for the evaluation of ligamentous wrist injuries, it is merely static and incapable of diagnosing dynamic wrist instability. Based on real-time MRI and algorithm-based image post-processing in terms of convolutional neural networks (CNNs), this study aims to develop and validate an automatic technique to quantify wrist movement. A total of 56 bilateral wrists (28 healthy volunteers) were imaged during continuous and alternating maximum ulnar and radial abduction. Following CNN-based automatic segmentations of carpal bone contours, scapholunate and lunotriquetral gap widths were quantified based on dedicated algorithms and as a function of wrist position. Automatic segmentations were in excellent agreement with manual reference segmentations performed by two radiologists as indicated by Dice similarity coefficients of 0.96 ± 0.02 and consistent and unskewed Bland–Altman plots. Clinical applicability of the framework was assessed in a patient with diagnosed scapholunate ligament injury. Considerable increases in scapholunate gap widths across the range-of-motion were found. In conclusion, the combination of real-time wrist MRI and the present framework provides a powerful diagnostic tool for dynamic assessment of wrist function and, if confirmed in clinical trials, dynamic carpal instability that may elude static assessment using clinical-standard imaging modalities.

## 1. Introduction

Intercarpal ligament injuries are frequent clinical entities, lead to malalignment of the carpal bones, and constitute the most common cause of carpal instability [1,2,3,4], thus increasing the risk of developing osteoarthritis [5,6]. To prevent irreversible joint damage, timely diagnosis of carpal instability is crucial.

Morphologic magnetic resonance imaging (MRI) is the most sensitive imaging modality for carpal soft tissue evaluation. While static carpal instability becomes evident in the neutral position and can be readily diagnosed using morphologic MRI, assessment of more complex carpal instability patterns that manifest only during active wrist movement remains difficult [7,8,9,10]. In clinical MRI exams, the wrist is immobilized to prevent movement artefacts and insufficient image quality and, consequently, imaged in a merely static configuration, which renders the diagnosis of complex carpal instability patterns impossible [1]. To overcome these diagnostic shortcomings, several dynamic and fast-imaging techniques have been introduced such as parallel imaging methods such as generalized autocalibrating partial parallel acquisition (GRAPPA) [11] and sensitivity encoding (SENSE) [12], under-sampling techniques combined with special image reconstruction pipelines such as k-t GRAPPA and k-t SENSE [13] as well as the so-called real-time MRI technique. These techniques enable very fast image acquisition, even of moving tissues and in real time [14,15,16], including active joint movement and joint kinematics [1]. For real-time MRI data acquisition, different sequences such as VIBE (volumetric interpolated breath-hold), as true fast imaging with steady state precession (TrueFISP) or highly under-sampled FLASH (fast low-angle shot) sequences with a radial encoding scheme, have been used for dynamic image acquisition [1,17,18]. However, as of today, only a few studies have reported the use of real-time MRI for the visualization of carpal kinematics. Recently, Shaw et al. assessed carpal instability using the VIBE sequence at a temporal resolution of 365 ms per image, which may not be sufficient to detect fine alterations in wrist movement. Even though in their study, the temporal resolution could be principally decreased to 135 ms, this acceleration was realized at the cost of the increased noise and decreased signal-to-noise ratio (SNR), which compromised the ability to accurately measure the scapholunate (SL) gap width [1]. Given the potential benefits of the FLASH sequence for real-time MRI—i.e., non-linear inverse reconstructions with temporal regularization that allows autocalibration during parallel imaging by simultaneously estimating coil sensitivities and image content [14]—it is principally possible to improve the temporal resolution to an effective 100 ms without time averaging, while upholding the spatial resolution [19]. This has been demonstrated in FLASH-based real-time MRI of the temporomandibular joint [17,18], aorta [20], and vocal tract during speech [21]. An overview of the basic principles of the MRI techniques cited previously has been added to appendix Table S1. In real-time MRI, the abundance of images acquired throughout the range-of-motion (ROM) brings about particular post-processing challenges. In the context of dynamic wrist evaluation at high temporal resolution, this necessarily entails tedious and labor-intensive measurements of inter-carpal joint gap widths as surrogates of carpal instability patterns. Consequently, there is a clear need to automatize carpal bone segmentations (that allow subsequent algorithm-based configurational measures to be derived) to position real-time MRI as a clinically useful technique. Convolutional neural networks (CNNs) lend themselves to such automatic segmentation tasks and have been applied in various medical fields by providing automatic segmentations of different entities and structures [22,23,24,25,26,27].

Against this background, our study’s objectives were to develop a CNN-based framework for automatic segmentation and quantification of wrist configuration during dynamic movement, to validate these measures against manual reference measures, and to demonstrate clinical applicability in volunteers and a patient. Our hypotheses were that (i) CNN-based segmentations of the carpus and forearm and their quantitative evaluation are as precise and accurate as manual reference measures, yet significantly faster, and that (ii) alterations in carpal bone configuration as a sign of complex carpal instability can be demonstrated in a patient based on the developed framework.

## 2. Materials and Methods

### 2.1. Study Design and Population

The study was designed as a descriptive observational in-vivo imaging study and approved by the local ethics committee (Ethics Committee, Faculty of Medicine, Heinrich-Heine-University Düsseldorf, Germany, study number 2019-590). Written informed consent was obtained from all participants prior to study initiation.

For the development of the framework, 56 bilateral wrists (28 healthy volunteers, mean age: 30.7 ± 13.6 years; age range 22 to 66 years; seven females, 21 males) were recruited among coworkers and students within the department and university. To assess principal clinical applicability following the framework’s thorough validation on healthy volunteers, a 42-year-old male patient with a partial SL ligament injury of the right hand as determined by morphologic MRI was included and assessed versus a size- and gender-matched healthy volunteer.

For the healthy volunteers, the exclusion criteria were as follows: history of surgery or trauma to the wrist, acute or chronic wrist pain, or established diagnosis of osteoarthritis, rheumatoid arthritis or other degenerative or inflammatory conditions of the upper extremity. Moreover, structural osseous and ligamentous integrity of the wrist and adjacent structures was assessed using morphologic MRI prior to the real-time MRI measurements as detailed below.

### 2.2. Image Data Acquisitions

MRI measurements were performed on a clinical 1.5-Tesla MRI scanner (MAGNETOM Avanto^fit^, Siemens Healthineers, Erlangen, Germany). All participants were positioned prone and head-forward with the imaged upper extremity outstretched above their head (“superman position”).

**Figure 1:**
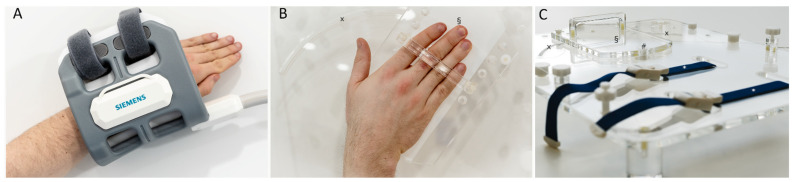
Coil and measurement setups during static reference and dynamic real-time MRI measurements. (**A**) For static measurements, a four-channel flex coil (Siemens Healthineers) was wrapped around the wrist to acquire the morphologic sequences. Shown are a left forearm and hand that were immobilized and imaged for reference purposes. (**B**) Positioning of a right wrist on the custom-made MRI-compatible movement device. The device has a mobile sliding tray (§) with a predefined range and trajectory of movement (x) onto which the hand was placed. (**C**) Tourniquets (*) were used to fix the forearm and prevent adaptive movement. The underside of the mobile sliding tray was coated with synthetic polytetrafluoroethylene (“Teflon”) to reduce friction. Centered underneath the wrist is the pivot point (#), which connects the mobile sliding tray and the fixed base plate. For dynamic measurements, a 24-channel spine matrix coil and an 18-channel body coil (both from Siemens Healthineers) were positioned underneath (spine matrix coil) and on top of the device (body coil) [not shown].

For static MRI, a four-channel flex coil (small, Siemens Healthineers) was wrapped around the wrist for optimized SNR (Figure 1A). All volunteers underwent clinical-standard MRI using T2-weighted (T2w) and proton density-weighted fat-saturated (PDw fs) sequences in the three main orientations (Table 1) to reliably rule out any pre-existing structural disorders of the wrists. D.B.A. (radiologist with five years of experience in musculoskeletal imaging) evaluated the sequences and confirmed the absence of relevant wrist pathologies in all volunteers.

For dynamic real-time MRI during active radial and ulnar deviation, a custom-made MRI-compatible movement device was used for radial and ulnar deviation during active wrist movement (Figure 1B,C). A 32-channel spine matrix coil (direct connect spine 32, Siemens Healthineers) underneath and an 18-channel body coil (Body 18, Siemens Healthineers) on top of the wrist-loaded device that was centrally positioned in the scanner’s bore were used for imaging. Individually adjustable spacers allowed the coils to be placed as close as possible to the wrist without restricting movement or being displaced through movement. Placing the coils as close to the wrist as possible was essential for a sufficiently high SNR. The wrist was loosely positioned on a sliding plate along the measurement plane and the sliding plate was guided by a circular full-thickness grooved trajectory that predefined the ROM from maximum ulnar abduction (30 to 35°) to maximum radial abduction (10 to 15°) and could be set individually by means of mechanical stoppers. To allow a frictionless movement, the underside of the sliding plate was coated with synthetic polytetrafluoroethylene (“Teflon”, DuPont, Wilmington, DE, USA). Adaptive forearm movement was prevented by two tourniquets attached to the movement device.

Practically, all participants were instructed to perform continuous and alternating radioulnar movement and to avoid any pronation, supination, flexion, or extension. Following out-of-scanner demonstrations, all participants trained to perform continuous movement before the actual measurements. During the acquisition period of the FLASH sequences of 30 s, each volunteer performed continuous radial to ulnar abduction and vice versa, i.e., waving, for approximately 15 s (for one full radial to ulnar abduction).

In preparation of this study, optimization of the radial FLASH real-time MRI sequence was performed to improve its temporal resolution while maintaining optimal SNR as well as bone and soft tissue contrast. After thorough optimization and validation [data not shown], sequence parameters of the real-time MRI sequence were selected as detailed in Table 1. Data acquisition was based on 21 radial spokes. These spokes were applied at complementary positions in five consecutive data sets. With the optimized real-time MRI sequence, a temporal resolution of approximately 95 ms (corresponding to 21 times TR = 4.5 ms) was realized without time averaging.

### 2.3. Manual Annotations and Reference Measurements

During each real-time FLASH sequence of 30 s, 300 images were acquired at a temporal resolution of approximately 95 ms per image. Consequently, only slight variations were expected between consecutive images. To facilitate manual segmentations of the wrist, the number of images was reduced considerably. To this end, all images acquired during one complete movement cycle, ranging from maximum radial abduction to maximum ulnar abduction, was selected by M.B. (radiologist with two years of experience in musculoskeletal imaging, reader 1) to be representative of the movement pattern of each volunteer and wrist. Of the entire image set (approximately 80 images), 15 individual images at equal temporal interval were selected for downstream analyses.

**Figure 2:**
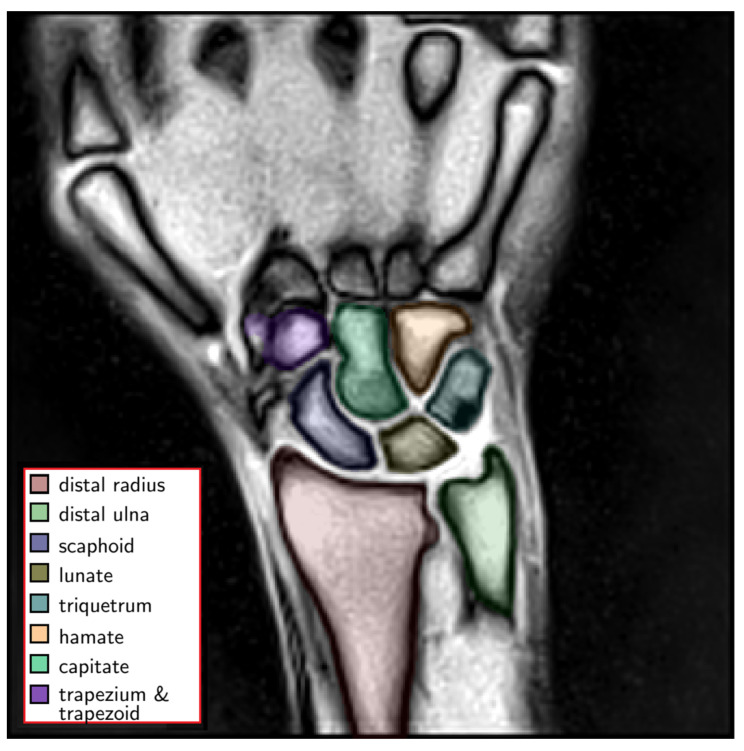
Exemplary color-coded manual segmentations of the wrist bones, i.e., carpal bones and distal forearm (left). These manual segmentations served as the ground truth for subsequent automatization. Uncolored anatomic structures are not part of the wrist and were considered as background.

A special graphical user interface, developed in-house and implemented in Python (v3.8.4, Python Software Foundation, Wilmington, DE, USA), was used for manual labeling and reference measurements by reader 1 and L.M.W. (radiologist with five years of experience in musculoskeletal imaging, reader 2). The following bone structures were segmented along their outer bone cortices: (1) distal radius, (2) distal ulna, (3) scaphoid, (4) lunate, (5) triquetrum, (6) hamate, (7) capitate, (8) trapezium and trapezoid (Figure 2) and served as ground truth.

In addition, wrist angle and SL and lunotriquetral (LT) gap widths were manually measured in each selected image along the entire ROM by both radiologists (reader 1 and reader 2). The wrist angle was determined by manually drawing two vectors with the first vector placed centrally along the forearm and wrist (to represent the central axis of radius, ulna, and carpus), and the second vector placed along the long axis of the capitate. The long axis of the capitate connected the centers of the proximal and distal poles of the capitate. In our study, radial deviation was defined by negative wrist angle values, while ulnar deviation was defined by positive values. The SL gap width was defined as the distance between the ulnar cortex of the scaphoid and the radial cortex of the lunate and measured at the centers of both bones between the proximal and distal articular surfaces along the proximal carpal row [1,8]. Accordingly, the LT gap width was defined as the central distance between the ulnar cortex of the lunate and the radial cortex of the triquetrum.

### 2.4. Framework for Measurements of Carpal Configurations during Dynamic Wrist movement

#### 2.4.1. Data Pre-Processing and Augmentation

For preparation of the image data, intensity normalization with adaptive window fitting based on the image histogram was performed. The 10th and 99th percentiles were determined for each image and the intensities were cropped above and below these thresholds. The images were then adjusted using the Z-score intensity normalization [28]. To increase spatial resolution for refined SL and LT gap definition, the MRI data were biquadratically interpolated from 168 × 168 pixels to 336 × 336 pixels using a Gaussian anti-aliasing filter. The size of the vector polygon masks was doubled accordingly.

The performance of deep neural networks directly correlates with the amount of data available for training [29]. Systematic data augmentation and variation by transforming the existing image datasets are important to improve the algorithm’s performance. To this end, the acquired images were subject to systematic noising (i.e., Gaussian noise σ ∈ [0, 0.2]), mirroring (along the *x*-axis), shifting (along the *x*-axis by up to ±10%), rotating (in the image plane by α ∈ [−15°, 15°]), and zooming (by up to 10% in and out). The manually defined segmentation masks were adjusted analogously based on the corresponding transformations. When zooming and rotating, the images were interpolated using the nearest-neighbour algorithm [30].

#### 2.4.2. Segmentation Framework

**Figure 3:**
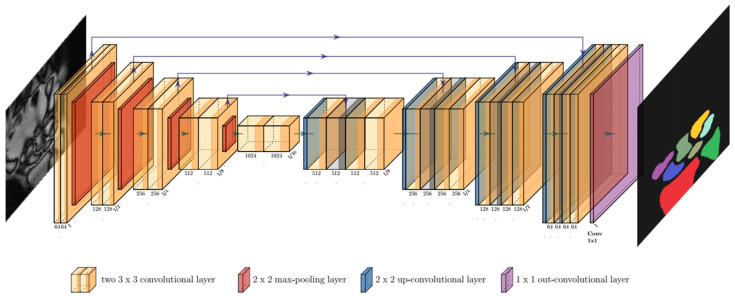
Convolution of an input MR image (left) inside the U-Net architecture and visualization of resultant bone segmentations as its output (right). The number of channels is indicated under each convolutional layer. The reduction and increase of the image resolution are indicated at the end of a block normalized to I = input size. Color-codes of the output image correspond to the different classes of semantic segmentations.

For the semantic segmentations of the carpus and forearm in each image, a dedicated framework was developed based on convolutional neural networks (CNNs). The structure of our CNN was based on a classic symmetric U-Net [31,32]. The contracting path (left side in Figure 3) consisted of repeated applications of two 3 × 3 convolutional layers, each followed by batch normalization and rectified linear unit layers. For down-sampling, a 2 × 2 max-pooling layer with a step size of 2 was used. The expanding path (right side in Figure 3) was symmetrically constructed. To fit the output of 64 feature vectors to the given number of eight classes, we used a 1 × 1 convolutional layer at the end.

#### 2.4.3. Post-Processing

Although U-Nets perform well in pixel-level segmentation, inaccurate segmentations may occur at the edges of the region-of-interest due to the loss of spatial information, i.e., spatial relations to the nearest neighbors, through the convolutions [32]. To improve the CNN’s ability to accurately segment the carpal bone cortices, a conditional random field (CRF) was applied. CRFs are widely used to combine the class values determined by the CNN with the low-level information (direct neighbor pixel information) [33,34]. Therefore, a CRF using mean-field approximation and convolutional approximation in the pairwise potential term was applied alongside the U-Net for semantic segmentation of the data.

The CNN-based segmentation framework segmented individual images independent of their k-nearest temporal neighbors. To further stabilize the segmentations, an additional post-processing algorithm was developed and implemented that corrected the segmentations of each pixel depending on four neighboring images (i.e., two images before and two images after the current image). To this end, pixel allocations to distinct classes following preliminary segmentations were weighted using Gaussian distributions of pixel allocations over time. Each pixel’s cumulative allocations over time ranged from 0 (pixel was not assigned to any class at any image over time) to 5 (pixel was assigned to a class at all time points), with the weighting favoring the influence of nearest temporal neighbors. Then, the mean value over time was calculated for each pixel. Using an empirical threshold of 0.6, any pixels above were assigned to the class, while any pixels below were assigned to the background. The calculation was performed separately for each image and class. Following the temporal post-processing, binary opening and closing operators (considering five connected pixels) were used to smooth the segmentation edges and fill holes within the segmentations [35]. Finally, only the pixels that formed the largest connected region for each class were included in the final segmentation mask. The dedicated post-processing strategy also stabilized the determination of the wrist angles (see Section 2.4.5) as it decreased sensitivity to isolated false-positive pixel segmentations and edges.

#### 2.4.4. Training and Evaluation

The developed framework was trained on a computer workstation with a Ryzen 9 3900X central processing unit (AMD, Santa Clara, CA, USA), 32 GB of main memory and a RTX 2070 Super graphics processing unit (NVIDIA, Santa Clara, CA, USA) with 8 GB of video random access memory. Training and evaluation routines were implemented in Python (v3.8.4) and the model was implemented in PyTorch, which is a library consisting of machine learning models [36]. For training, the Adam optimizer [37] was employed using an initial learning rate of 0.1 and a weight decay of 1 × 10^−8^. During training, the learning rates were reduced by gamma 0.1 using multi-step scheduling. The model was trained over 500 epochs with a batch size of 4 and the milestones of multi-step scheduling were set at epochs 10, 50, 100, 150 and 250. Image datasets of 42 bilateral wrists (i.e., 21 volunteers) were used for training, while image datasets of 14 bilateral wrists (i.e., seven volunteers) were used for testing. Based on the 15 selected images per wrist (as detailed above), the training and test datasets consisted of 630 images and 210 images, respectively.

Using the dice similarity coefficient (DSC) [38] that quantifies similarity between two samples and ranges between 0 (i.e., no overlap) and 1 (i.e., complete overlap), our framework’s segmentation performance was evaluated against the manual reference segmentations. A sum loss function of the dice error function [39] and the Hausdorff distance loss function [40] were used.

#### 2.4.5. Algorithm-Based Measurements of Carpal Configurations

**Figure 4:**
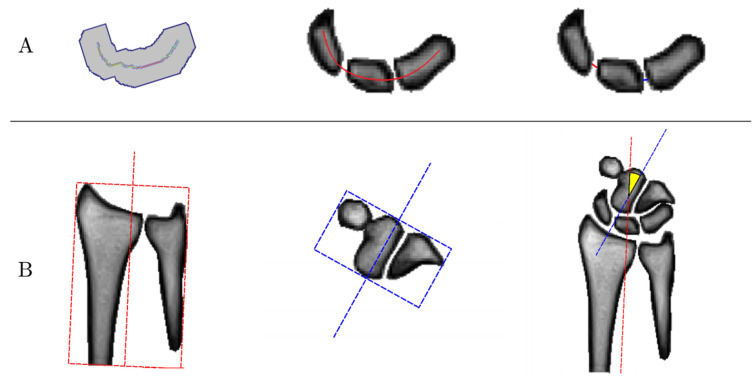
Graphical visualization of the algorithm-based measurements of carpal configuration. (**A**) Determination of scapholunate (SL) and lunotriquetral (LT) gap widths. First, the bone masks of the scaphoid, lunate, and triquetrum were connected (left). Then, the centerline of the three bones was determined along the carpal arcs (center). Eventually, the SL (red) and LT (blue) gap widths were determined based on the intersections of this centerline and the bone contours. (**B**) Determination of the wrist angle. The minimum bounding boxes around the bone contours of the forearm, i.e., radius and ulna (red, left), as well as around the distal row of the carpal bones (blue, center) were determined. Then, the wrist angle was calculated as the angle between the centers of the two boxes (yellow, right).

Based on the automatic segmentations of our framework, the diagnostic measures SL and LT gap widths were derived as a function of wrist angle. Methodologically, measurement of the SL and LT gap widths and the wrist angle were implemented in close emulation of the manual reference measurements (see Section 2.4.2). First, the bone masks of the scaphoid, lunate, and triquetrum were smoothed and connected for better generalization. Second, the centerline along the scaphoid, lunate, and triquetrum was determined along the carpal arcs of the proximal convexities and distal concavities of these structures [41]. Third, the distances between the intersections of this centerline with the ulnar cortex of the scaphoid and the radial cortex of the lunate was defined as the SL gap width, while the distances between the intersections of this centerline with the ulnar cortex of the lunate and the radial cortex of the triquetrum was defined as the LT gap width (Figure 4A). Fourth, to calculate the wrist angle, the minimum bounding boxes around the segmentation masks of the distal row of the carpal bones as well as the radius and the ulna were determined. The wrist angle was then determined between the centers of the two bounding boxes (Figure 4B).

### 2.5. Statistical Analysis

Statistical analyzes were performed in R software (v4.0.3, R Foundation for Statistical Computing) by K.L.R. For the calculation of the linear mixed model (LMM) as detailed below, the statistical software SPSS (v27, SPSS Inc., Chicago, IL, USA) was used.

Descriptive statistics for the SL and LT gap widths, wrist angles, and DSCs were calculated for all volunteers. Unless otherwise specified, data are given as means ± standard deviations (SDs).

To evaluate segmentation performance, the following comparisons were made: framework vs. manual segmentation 1 (reader 1), framework vs. manual segmentation 2 (reader 2), and manual segmentation 1 vs. manual segmentation 2.

Bland–Altman plots were used to visualize and comparatively evaluate automatically and manually determined SL and LT gap widths as well as wrist angles.

An LMM was used to evaluate the SL and LT gap widths based on multivariable statistics. The model included a subject-specific factor, the factors gender and wrist side, and the covariates age and height. The measurement repetition of each hand was calculated using the first-order autoregressive moving average model [42]. The model was fitted using the constrained maximum likelihood approach [43,44].

Due to the exploratory design of this study and the large amount of statistically relevant data, the significance level was set to *p* ≤ 0.01. In addition, alpha error accumulation was countered using the strict Bonferroni correction. This “lower-than-usual” significance level was chosen to prevent inflation of the alpha error while maintaining statistical power and reducing the false-negative rate.

## 3. Results

### 3.1. Segmentation Performance

Automatic segmentations of the carpus and forearm were in excellent agreement with the manual reference segmentations. The mean DSCs across the eight classes were 0.966 ± 0.017 (automatic vs. reader 1) and 0.953 ± 0.016 (automatic vs. reader 2), respectively (Table 2). Overall, DSC values between training and test datasets were closely related, indicating the framework’s sufficient generalization. In comparison, the mean inter-reader DSC was 0.962 ± 0.018 (reader 1 vs. reader 2). Quantitative inter-reader and inter-method similarity in terms of DSC values is detailed as the function of class, i.e., bone, and pair-wise comparison in Table 2.

Evaluation of a complete real-time MRI sequence of 30 s duration that consisted of 300 images—i.e., automatic segmentation and determination of the SL and LT gap widths and wrist angles—required 1:23 min based on the hardware configuration as detailed above.

### 3.2. Inter-Method and Inter-Reader Reliability Analysis

**Figure 5:**
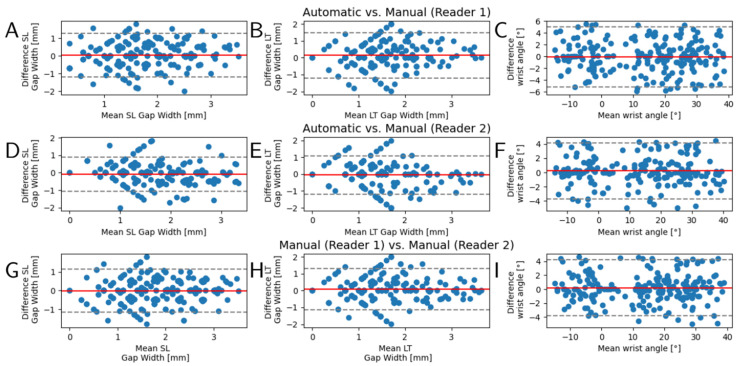
Bland–Altman plots to evaluate inter-method and inter-reader reliability. Automatic segmentations and subsequently determined diagnostic measures, i.e., scapholunate (SL) gap width (**A**,**D**,**G**), lunotriquetral (LT) gap width (**B**,**E**,**H**), and wrist angle (**C**,**F**,**I**), were evaluated against the manual reference measurements by two radiologists (reader 1, reader 2). Y-axes indicate the respective measures’ inter-method or inter-reader differences, while x-axes indicate the mean measure. Visualized are the 210 values of the test datasets as derived from 15 representative images across the entire range-of-motion. Red lines indicate the mean of the differences and gray dashed lines the 95% confidence intervals.

**Figure 6:**
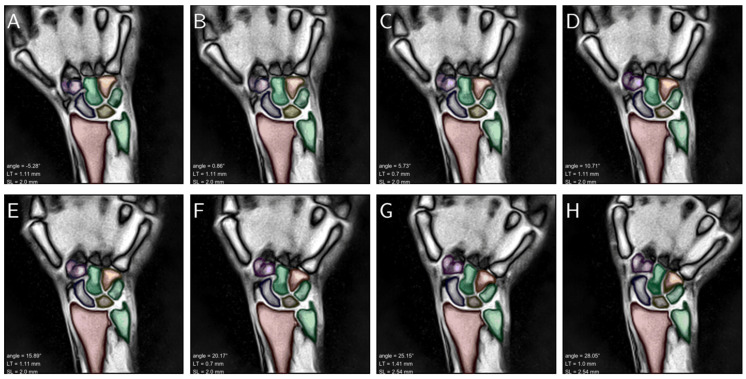
Consecutive still images of a left wrist across the entire range of active radioulnar movement that ranged from maximum radial abduction to maximum ulnar abduction (**A**–**H**). The carpus and forearm are color-coded as in Figure 2 and overlaid onto the morphologic images. Inset (lower left) are the resultant wrist angles (“angle”), LT gap width (“LT”), and SL gap width (“SL”). The corresponding video that visualizes fluent movement across the entire range-of-motion is appended as Video S1.

Bland–Altman plots were generated to systematically and comparatively evaluate inter-method and inter-reader reliability in the quantification of the diagnostic measures, i.e., SL and LT gap widths and wrist angle, on the test dataset. The distributions of measurement differences between readers and the framework were found to be largely balanced. No systematic differences or outliers were detected and the mean differences were determined as 0 or close to 0 (Figure 5). Qualitative evaluation of segmentation accuracy and coherence across the entire ROM was correct and is exemplified in Figure 6 and Video S1. The derived clinical measures SL and LT gap widths as well as wrist angles were plausible, too.

### 3.3. Multivariable Comparative Analyses of the SL and LT Gap Widths

Based on the LMM, we found that in the healthy volunteers, the factors wrist angle, side, and height significantly influenced the SL or LT gap widths, respectively, across the ROM (Table S2). For the comparisons of wrist side and gender, the covariances were corrected to their mean (wrist angle: 9.65°, height: 1.79 m, age: 30.74 years). The calculations were based on the fully automated analyses of our framework. In total, 16,800 real-time MR images (56 wrists from 28 volunteers) were analyzed. In the following, the mean values refer to the estimates of the LMM; therefore, the standard error is given instead of the standard deviation. Furthermore, the 99% confidence intervals (CI) refer to the distribution of the estimated means.

For the SL gap width, we found significant associations with the factors side (*p* < 0.001) and wrist angle (*p* = 0.002.) (Table S2). The estimated mean SL gap width was 1.25 mm (99% CI 1.12; 1.40) for the left hand and 1.88 mm (99% CI 1.75; 2.02) for the right hand (Table 3). For the covariate wrist angle, the estimated fixed parameter was −0.002 ± 0.001 mm/degree (Table S1); therefore, we found a slight but significant decrease of the SL gap during ulnar abduction. No significant dependence on the factors gender, height or age could be observed (Table S2).

For the LT gap width, there was a significant influence of wrist angle (*p* < 0.001) and body height (*p* < 0.001). The fixed parameters were −0.003 ± 0.001 mm/degree for the wrist angle and 0.019 ± 0.005 mm/cm for the body height. In contrast, the factors gender, side, and age had no significant influence (Table 3 and Table S2).

### 3.4. Assessment of Clinical Applicability in a Patient

**Figure 7:**
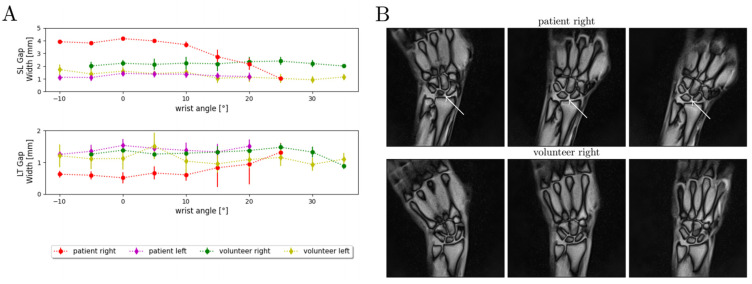
Quantitative and qualitative changes in carpal configurations in a patient with a partial scapholunate ligament rupture and a gender- and size-matched healthy volunteer during active radioulnar movement. (**A**) SL and LT gap widths are given as a function of wrist angle across the range-of-motion. The gap widths are indicated as means (dots) and standard deviations (whiskers). A total of 300 MR images per wrist were analyzed and quantified by our framework. Indicated are the measured SL and LT gap widths of the wrist-injured patient (partial SL ligament rupture, “patient right”, red), the contralateral wrist (“patient left”, purple), and the corresponding wrists of the matched and healthy volunteer (“volunteer right”, green; “volunteer left”, yellow). For the sake of visualization, the wrist angles are grouped at intervals of 5°. (**B**) Three exemplary MR images are shown for the patient and the volunteer at various positions throughout the active radioulnar movement range. The increased dehiscence of the SL gap of the injured wrist (white arrows) is particularly obvious in the neutral position and ulnar abduction and at the proximal portion of the SL gap.

To demonstrate clinical applicability of the developed framework, SL and LT gap widths were assessed as a function of ROM in a patient with an established diagnosis of SL ligament injury (male, 42 years) and compared with a gender- and height-matched healthy volunteer (male, 42 years). The patient had sustained a partial SL ligament injury (dorsal component of the SL ligament) of his right wrist four years earlier and was still symptomatic with pain under stress and subjectively decreased ROM. Despite his symptoms, he was able to move his wrist continuously in the device. For ligament-intact wrists (i.e., left wrist (patient), both wrists (volunteer)), limited variability in SL gap width was observed as a function of wrist angle and along the entire ROM with stable SL and LT gap widths of approximately 1.5 to 2 mm and 1 to 1.5 mm, respectively (Figure 7A). In contrast, the patient’s injured wrist exhibited SL and LT gap widths that changed considerably with wrist angle, in particular in pronounced ulnar abduction. While between 10° of radial abduction and 10° of ulnar abduction, the SL and LT gap widths remained about constant at approximately 4 mm and 0.7 mm, respectively; the gap widths decreased to 1 mm (SL gap width) and increased above 1 mm (LT gap width) with further ulnar abduction (Figure 7A). In addition, the injured wrist’s ulnar abduction was decreased compared to the contralateral side (Figure 7A). Alongside dynamic changes of the carpal configuration, constitutively higher SL and lower LT gaps were demonstrated throughout the entire active ROM when compared to the matched healthy wrist (Figure 7B).

## 4. Discussion

In our study, we could successfully image, process, analyze, and quantify active radioulnar wrist movement to automatically determine the SL and LT gap widths as a function of wrist angle using CNN-based semantic segmentation of the carpus and forearm. With a total acquisition time of the real-time FLASH MRI sequence of only 30 s, sufficient image data of active wrist movement could be acquired to derive diagnostic measures. In addition, a dedicated post-processing pipeline was implemented based on a cohort of healthy volunteers and demonstrated to be applicable in a patient with SL ligament injury. With the diagnostic capabilities of morphologic MRI limited in the detection of complex carpal instability patterns, there is a clinical need for dynamic imaging modalities. Wrist cinematography assesses altered carpal kinematics in patients with suspected SL injury, yet the use of ionizing radiation, lack of standardization, and inability to visualize soft tissues limit its clinical application [9]. Our approach systematically improved dynamic imaging of the wrist based on real-time MRI with regards to image acquisition and post processing. This was realized by bringing together an optimized morphologic FLASH sequence with high temporal resolution, standardized active radioulnar movement of the wrist, and sophisticated CNN-based segmentation and algorithmic determination of diagnostic measures. However, the high temporal resolution resulted in more than 10 images being acquired per second, which rendered manual segmentation and evaluation impractical, even in controlled research contexts, and necessitated further automatization. As suggested by earlier studies that indicated the potential of CNNs in the automated and reliable segmentation of clinical MRI datasets in recent years [27,28,45,46], this study confirmed the high accuracy and reliability of CNN-based semantic segmentations in the context of dynamic wrist imaging.

Previous studies reported that radial sampling schemes are less sensitive to movement artifacts (due to the inherent averaging of low spatial frequencies) and are therefore well suited for the assessment of active movement [1,8]. In a recent study by Shaw et al. [1], real-time MRI was used to assess dynamic changes in carpal configuration during active movement. Using a temporal resolution of 315 ms, Shaw et al. proved that accurate manual measurements of diagnostic measures of carpal configurations were feasible. However, while their gradient-echo sequence’s temporal resolution could be reduced to 135 ms per image, this acceleration came at the cost of similarly reduced image quality that may compromise the ability to accurately measure carpal configurations. Our optimized real-time radial FLASH sequence allows the quantification of the SL and LT gap width using a temporal resolution of approximately 95 ms. This acceleration did not induce a relevant loss of image quality and, consequently, the CNN-based post-processing quantification technique was able to segment and evaluate all acquired images without additional user input. Unlike other real-time MRI techniques, the FLASH sequence used in our study is characterized by “true” temporal resolution, which means that the images are determined based on the nonlinear inverse reconstructions without temporal averaging. Other real-time MRI techniques are based on the “sliding window technique”, which means that the k-space lines are only partially updated for spatial resolution. Consequently, the high spatial resolution is thus ensured by temporal averaging; the true temporal resolution is therefore considerably longer. The FLASH sequence, on the other hand, is based on a nonlinear inverse reconstruction technique; thus, the high temporal resolution is not lost [19]. Our framework requires 1:23 min for complete processing of a complete real-time MRI sequence of 30 s duration. Thus, the post-processing pipeline is unlikely to lead to any significant delays if used in the clinical routine, and is thus well poised to be of diagnostic assistance.

Our framework is also characterized by an excellent segmentation performance with mean DSC values of ≥0.96. Bland–Altman plots showed no systematic and method-related differences when comparing manual and automatic measurements. Based on the LMM, 28 healthy volunteers were compared for SL and LT gap width; here, the estimated mean value for SL gap width was 1.52 mm in males (99% CI 1.41, 1.62) and 1.62 in females (99% CI 1.38, 1.86), which is within the range published in the literature [47]. In comparison, the SL gap width increased in a patient with a partial SL ligament rupture, which is consistent with other literature data [9,48]. In addition, the patient showed a significantly stronger dependence of the SL and LT gap width on wrist angle, i.e., hand position, as compared with the matched healthy volunteer. Furthermore, we observed significantly (*p* < 0.001) smaller mean SL gap widths for the left side (1.26 mm [99% CI 1.12, 1.40]) than the right side (1.88 mm (99% CI 1.75, 2.02)). Even though the exact reason for this difference remains speculative, it may involve the handedness of the volunteers that, unfortunately, had not been registered in this study and remains to be evaluated in larger clinical trials.

Although we found scientifically and clinically encouraging results, some limitations should be mentioned. First, our optimized real-time MRI framework for image acquisition and post-processing can only indirectly assess ligament integrity. While direct imaging of the SL ligament has already been performed using longer repetition times [49,50], this modification would decrease temporal resolution, and thus could possibly compromise the evaluation of dynamic wrist movement. Second, clinical use of real-time MRI may be limited in patients with posttraumatic limitations of mobility due to pain, swelling, tension, or haemarthrosis so that indications may be limited to more chronic cases. Due to the inability to directly assess the carpal soft tissues as outlined above, the current real-time MRI technique may not visualize ligament tears or strains or other non-bony stabilizers of the wrist. Third, our healthy wrist cohort was relatively young and may not be representative of the full age spectrum of clinically relevant wrist pathologies. Fourth, our multivariable analyses were based on an LMM and assumed that the dependent variables SL and LT gap widths behave linearly with the fixed factors and covariates. However, due to the small number of participants, it is not possible to compare different higher-order models with respect to their predictive performance. In addition, only the main factors were compared; whether these factors are further conditional on each other was not analyzed. Fifth, in patients with advanced carpal instability, aberrant rotational movement of the carpus during active movement could complicate the automatic segmentation and algorithm-based measurements. While real-time MRI techniques that cover larger volumes have already been implemented [1], such volumetric approaches come along with lower spatial and temporal resolutions and limitations in SNR. For clinical use, it may thus be beneficial to increase the number of slices measured so that the technique is more robust to out-of-plane bone movement. Sixth, the framework we implemented only acquires various clinical parameters. However, previous studies have shown that systems such as support vector machines and CNNs can be used to automatically classify the extracted features in terms of healthy and diseased and in terms of different clinical conditions, respectively [51,52]. In the study by Dachena et al., such systems achieved over 95% accuracy [52]. In follow-up studies, it is therefore of interest to investigate different clinical conditions of carpal instability and to divide them into groups with respect to their features. Seventh, our proposed framework for assessing complex carpal instability has been demonstrated on one patient only with static carpal instability that had been diagnosed with clinical-standard static MRI. It remains to be seen in larger clinical studies whether the framework can be used to assess more complex instability patterns including dynamic carpal instability. Eighth, to sufficiently enable SNR for our MR measurements at 1.5 Tesla, 21 spokes were acquired per image. Increasing the field strength would enable an increase of SNR or an acceleration of acquisition speed. For example, Krohn et al. was able to acquire real-time images of the temporomandibular joint at 3 Tesla with 11 spokes per image [18].

## 5. Conclusions

The combination of advanced image acquisition and post-processing techniques renders dynamic wrist assessment across the entire range of radioulnar active movement feasible, both in healthy volunteers and ligament-injured patients. By bringing together standardized real-time MRI of the wrist at high temporal resolution with maintained spatial resolution and a CNN-based semantic segmentation approach with subsequent quantification of carpal configuration (in terms of SL and LT gap widths as a function of wrist angle), our framework provides a powerful diagnostic tool for dynamic assessment of wrist function. If confirmed in clinical trials, more complex carpal instability patterns that may elude static assessment using clinical-standard morphologic MRI may eventually be assessable non-invasively and without ionizing radiation.

## Figures and Tables

**Table 1:** Acquisition parameters of MR sequences. For the PD-weighted fat-saturated sequences, duration refers to the acquisition of a single orientation. Total measurement time for the acquisition of the three orientations was 8:42 min.

	T2-Weighted	PD-Weighted fs	Radial Real-Time
Sequence type	TSE	TSE	FLASH
Turbo Factor	10	10	n.a.
Orientation	ax	cor, ax, sag	cor
Repetition time (ms)	5820	3200	4.5
Echo time (ms)	114	33	2.5
Field of View (mm)	150 × 150	120 × 120	168 × 168
Image matrix (pixels)	384 × 384	256 × 256	168 × 168
Pixel size (mm/pixel)	0.4 × 0.4	0.5 × 0.5	1.0 × 1.0
Flip angle (°)	15	180	7
Slices (n)	20	20	1
Slice thickness (mm)	3	2	6
Examination time (sec)	194	174	30

Abbreviations: cor—coronal, ax—axial, sag—sagittal, PD—proton density, fs—fat-saturated, TSE—turbospin-echo, FLASH—fast low-angle shot, n.a.—not applicable.

**Table 2:** Similarity of manual and automatic segmentations as indicated by dice similarity coefficients (DSC, mean ± standard deviation) for each of eight classes, i.e., anatomic forearm or carpal bones. Manual reference segmentations were performed by two radiologists, i.e., reader 1 and reader 2.

	Reader 1 vs. Reader 2		Automatic vs. Reader 1		Automatic vs. Reader 2	
	Training Data	Test Data	Training Data	Test Data	Training Data	Test Data
Distal Radius	0.962 ± 0.060	0.972 ± 0.014	0.968 ± 0.061	0.969 ± 0.054	0.964 ± 0.025	0.957 ± 0.057
Distal Ulna	0.952 ± 0.044	0.954 ± 0.025	0.960 ± 0.039	0.957 ± 0.056	0.948 ± 0.029	0.964 ± 0.025
Scaphoid	0.964 ± 0.019	0.957 ± 0.022	0.970 ± 0.020	0.970 ± 0.022	0.952 ± 0.020	0.952 ± 0.024
Lunate	0.966 ± 0.016	0.964 ± 0.013	0.975 ± 0.014	0.972 ± 0.040	0.958 ± 0.018	0.954 ± 0.040
Triquetrum	0.964 ± 0.021	0.960 ± 0.019	0.969 ± 0.018	0.970 ± 0.021	0.955 ± 0.017	0.950 ± 0.026
Hamate	0.966 ± 0.018	0.964 ± 0.016	0.968 ± 0.021	0.971 ± 0.015	0.955 ± 0.022	0.956 ± 0.018
Capitate	0.969 ± 0.014	0.969 ± 0.011	0.976 ± 0.016	0.977 ± 0.017	0.963 ± 0.017	0.963 ± 0.017
Trapezium and Trapezoid	0.960 ± 0.027	0.942 ± 0.035	0.953 ± 0.026	0.924 ± 0.048	0.942 ± 0.024	0.919 ± 0.044

**Table 3:** Estimated mean values of scapholunate (SL) and lunotriquetral (LT) gap widths (mm) as a function of gender and side based on a linear mixed model. The model included the fixed effects gender and side as well as the covariates wrist angle, height, and age. For the calculation, 16,800 real-time MR images of 56 wrists (28 healthy volunteers) were included.

	Factors	Subfactors	Model Estimates ^1^		
			Mean Value	99% Confidence Interval	
SL gap width	Gender	male	1.52	1.41	1.62
		female	1.62	1.38	1.86
	Side	right	1.88	1.75	2.02
		left	1.26	1.12	1.40
LT gap width	Gender	male	1.28	1.19	1.37
		female	1.42	1.22	1.63
	Side	right	1.37	1.25	1.49
		left	1.33	1.22	1.45

^1^ The covariates were corrected for a wrist angle of 9.65°, a height of 1.79 m, and an age of 30.74 years.

## Data Availability

Data can be provided by the authors upon reasonable request.

## Supplementary Materials

The following are available online at https://www.mdpi.com/article/10.3390/diagnostics11061077/s1, Table S1: overview of a selection of fast MR sequences with a short description of the basic principle, Table S2: summary of the linear mixed model; Video S1: continuous alternating active radioulnar abductions (“waving”) of the left wrist of a healthy volunteer as imaged and processed by the framework.

## References

[1] Shaw C.B., Foster B.H., Borgese M., Boutin R.D., Bateni C., Boonsri P., Bayne C.O., Szabo R.M., Nayak K.S., Chaudhari A.J. (2019). Real-time three-dimensional MRI for the assessment of dynamic carpal instability. PLoS ONE.

[2] Schernberg F. (1990). Roentgenographic examination of the wrist: A systematic study of the normal, lax and injured wrist Part 2: Stress views. J. Hand Surg..

[3] Moser T., Dosch J.-C., Moussaoui A., Dietemann J.-L. (2007). Wrist Ligament Tears: Evaluation of MRI and Combined MDCT and MR Arthrography. Am. J. Roentgenol..

[4] Theumann N.H., Etechami G., Duvoisin B., Wintermark M., Schnyder P., Favarger N., Gilula L.A. (2006). Association between Extrinsic and Intrinsic Carpal Ligament Injuries at MR Arthrography and Carpal Instability at Radiography: Initial Observations. Radiology.

[5] Watson H.K., Ballet F.L. (1984). The SLAC wrist: Scapholunate advanced collapse pattern of degenerative arthritis. J. Hand Surg..

[6] Kiefhaber T.R. (2009). Management of Scapholunate Advanced Collapse Pattern of Degenerative Arthritis of the Wrist. J. Hand Surg..

[7] Taleisnik J. (1988). Current concepts review. Carpal instability. J. Bone Jt. Surg. Am. Vol..

[8] Boutin R.D., Buonocore M.H., Immerman I., Ashwell Z., Sonico G.J., Szabo R.M., Chaudhari A.J. (2013). Real-Time Magnetic Resonance Imaging (MRI) during Active Wrist motion—Initial Observations. PLoS ONE.

[9] Manuel J., Moran S.L. (2007). The Diagnosis and Treatment of Scapholunate Instability. Orthop. Clin. N. Am..

[10] Ramamurthy N.K., Chojnowski A.J., Toms A.P. (2015). Imaging in carpal instability. J. Hand Surg..

[11] Griswold M.A., Jakob P.M., Heidemann R., Nittka M., Jellus V., Wang J., Kiefer B., Haase A. (2002). Generalized autocalibrating partially parallel acquisitions (GRAPPA). Magn. Reson. Med..

[12] Pruessmann K.P., Weiger M., Scheidegger M.B., Boesiger P. (1999). SENSE: Sensitivity encoding for fast MRI. Magn. Reson. Med..

[13] Tsao J., Boesiger P., Pruessmann K.P. (2003). K-T BLAST and K-T SENSE: Dynamic MRI with high frame rate exploiting spatiotemporal correlations. Magn. Reson. Med..

[14] Uecker M., Zhang S., Voit D., Karaus A., Merboldt K.-D., Frahm J. (2010). Real-time MRI at a resolution of 20 ms. NMR Biomed..

[15] Feng L., Tyagi N., Otazo R. (2020). MRSIGMA: Magnetic Resonance SIGnature MAtching for real-time volumetric imaging. Magn. Reson. Med..

[16] van Amerom J.F., Lloyd D.F., Deprez M., Price A.N., Malik S.J., Pushparajah K., van Poppel M.P., Rutherford M.A., Razavi R., Hajnal J.V. (2019). Fetal whole-heart 4D imaging using motion-corrected multi-planar real-time MRI. Magn. Reson. Med..

[17] Krohn S., Gersdorff N., Wassmann T., Merboldt K.-D., Joseph A.A., Buergers R., Frahm J. (2016). Real-time MRI of the temporomandibular joint at 15 frames per second—A feasibility study. Eur. J. Radiol..

[18] Krohn S., Joseph A.A., Voit D., Michaelis T., Merboldt K.-D., Buergers R., Frahm J. (2019). Multi-slice real-time MRI of temporomandibular joint dynamics. Dentomaxillofac. Radiol..

[19] Frahm J., Schätz S., Untenberger M., Zhang S., Voit D., Merboldt K.D., Sohns J.M., Lotz J., Uecker M. (2014). On the Temporal Fidelity of Nonlinear Inverse Reconstructions for Real-Time MRI—The motion Challenge. Open Med. Imaging J..

[20] Joseph A., Kowallick J.T., Merboldt K., Voit D., Schaetz S., Zhang S., Sohns J.M., Lotz J., Frahm J. (2014). Real-time flow MRI of the aorta at a resolution of 40 msec. J. Magn. Reson. Imaging.

[21] Niebergall A., Zhang S., Kunay E., Keydana G., Job M., Uecker M., Frahm J. (2012). Real-time MRI of speaking at a resolution of 33 ms: Undersampled radial FLASH with nonlinear inverse reconstruction. Magn. Reson. Med..

[22] Pereira S., Pinto A., Alves V., Silva C.A. (2016). Brain Tumor Segmentation Using Convolutional Neural Networks in MRI Images. IEEE Trans. Med. Imaging.

[23] Wu B., Fang Y., Lai X. (2020). Left ventricle automatic segmentation in cardiac MRI using a combined CNN and U-net approach. Comput. Med. Imaging Graph..

[24] Essa E., Aldesouky D., Hussein S., Rashad M.Z. (2020). Neuro-fuzzy patch-wise R-CNN for multiple sclerosis segmentation. Med. Biol. Eng. Comput..

[25] Liu F., Zhou Z., Jang H., Samsonov A., Zhao G., Kijowski R. (2018). Deep convolutional neural network and 3D deformable approach for tissue segmentation in musculoskeletal magnetic resonance imaging. Magn. Reson. Med..

[26] Brui E., Efimtcev A.Y., Fokin V.A., Fernandez R., Levchuk A., Ogier A.C., Samsonov A.A., Mattei J.P., Melchakova I.V., Bendahan D. (2020). Deep learning-based fully automatic segmentation of wrist cartilage in MR images. NMR Biomed..

[27] Schock J., Truhn D., Abrar D.B., Merhof D., Conrad S., Post M., Mittelstrass F., Kuhl C., Nebelung S. (2021). Automated Analysis of Alignment in Long-Leg Radiographs by Using a Fully Automated Support System Based on Artificial Intelligence. Radiol. Artif. Intell..

[28] Buda M., Saha A., Mazurowski M.A. (2019). Association of genomic subtypes of lower-grade gliomas with shape features automatically extracted by a deep learning algorithm. Comput. Biol. Med..

[29] Khan Z., Yahya N., Alsaih K., Ali S.S.A., Meriaudeau F. (2020). Evaluation of Deep Neural Networks for Semantic Segmentation of Prostate in T2W MRI. Sensors.

[30] George W. (1994). Digital Image Warping.

[31] Ronneberger O., Fischer P., Brox T. (2015). U-Net: Convolutional Networks for Biomedical Image Segmentation. Lecture Notes in Computer Science.

[32] Zhang J., Li C., Kulwa F., Zhao X., Sun C., Li Z., Jiang T., Li H., Qi S. (2020). A Multiscale CNN-CRF Framework for Environmental Microorganism Image Segmentation. BioMed Res. Int..

[33] Zheng S., Jayasumana S., Romera-Paredes B., Vineet V., Su Z., Du D., Huang C., Torr P.H.S. Conditional Random Fields as Recurrent Neural Networks. Proceedings of the 2015 IEEE International Conference on Computer Vision (ICCV).

[34] Chen L.-C., Papandreou G., Kokkinos I., Murphy K., Yuille A.L. (2017). DeepLab: Semantic Image Segmentation with Deep Convolutional Nets, Atrous Convolution, and Fully Connected CRFs. IEEE Trans. Pattern Anal. Mach. Intell..

[35] Moldovanu S., Moraru L., Biswas A. (2015). Robust Skull-Stripping Segmentation Based on Irrational Mask for Magnetic Resonance Brain Images. J. Digit. Imaging.

[36] Paszke A., Gross S., Massa F., Lerer A., Bradbury J., Chanan G., Killeen T., Lin Z., Gimelshein N., Antia L. (2019). PyTorch: An Imperative Style, High-Performance Deep Learning Library.

[37] Kingma D.P., Ba J. (2015). Adam: A Method for Stochastic Optimization. Published as a conference paper at the 3rd International Conference for Learning Representations, San Diego, California, USA. arXiv.

[38] Jadon S. A survey of loss functions for semantic segmentation. Proceedings of the 2020 IEEE Conference on Computational Intelligence in Bioinformatics and Computational Biology (CIBCB).

[39] Sudre C.H., Li W., Vercauteren T., Ourselin S., Cardoso M.J. (2017). Generalised Dice Overlap as a Deep Learning Loss Function for Highly Unbalanced Segmentations. Deep Learning in Medical Image Analysis and Multimodal Learning for Clinical Decision Support.

[40] Ribera J., Güera D., Chen Y., Delp E. (2018). Weighted Hausdorff Distance: A Loss Function for Object Localization. arXiv.

[41] Lee T.C., Kashyap R.L., Chu C.N. (1994). Building Skeleton Models via 3-D Medial Surface Axis Thinning Algorithms. CVGIP Graph. Models Image Process..

[42] (2011). Autoregressive Moving Average Models. Time Series.

[43] Zhu S., Wathen A.J. (2018). Essential formulae for restricted maximum likelihood and its derivatives associated with the linear mixed models. arXiv.

[44] Beaumont C. (1991). Comparison of Henderson\textquotesingles Method I and Restricted Maximum Likelihood Estimation of Genetic Parameters of Reproductive Traits. Poult. Sci..

[45] Meyer A., Chlebus G., Schreiber A., Hansen C., Rak M., Schindele D., Schostak M., van Ginneken B., Schenk A., Meine H. (2021). Anisotropic 3D Multi-Stream CNN for Accurate Prostate Segmentation from Multi-Planar MRI. Comput. Methods Programs Biomed..

[46] Belal S.L., Sadik M., Kaboteh R., Enqvist O., Ulén J., Poulsen M.H., Simonsen J., Høilund-Carlsen P.F., Edenbrandt L., Trägårdh E. (2019). Deep learning for segmentation of 49 selected bones in CT scans: First step in automated PET/CT-based 3D quantification of skeletal metastases. Eur. J. Radiol..

[47] Andersson J.K. (2017). Treatment of scapholunate ligament injury. EFORT Open Rev..

[48] Chennagiri R.J.R., Lindau T.R., Chennagiri R.J.R., Lindau T.R. (2013). Assessment of scapholunate instability and review of evidence for management in the absence of arthritis. J. Hand Surg..

[49] Spaans A.J., Van Minnen P., Prins H.J., Korteweg M.A., Schuurman A.H. (2013). The Value of 3.0-Tesla MRI in Diagnosing Scapholunate Ligament Injury. J. Wrist Surg..

[50] Greditzer H.G., Kam C.C., Gray R.R., Clifford P.D., Mintz D.N., Jose J., Zeidenberg J. (2016). Optimal detection of scapholunate ligament tears with MRI. Acta Radiol..

[51] Zhou H., Hallac R.R., Yuan Q., Ding Y., Zhang Z., Xie X.-J., Francis F., Roehrborn C.G., Sims R.D., Costa D.N. (2017). Incorporating Oxygen-Enhanced MRI into Multi-Parametric Assessment of Human Prostate Cancer. Diagnostics.

[52] Dachena C., Casu S., Fanti A., Lodi M.B., Mazzarella G. (2019). Combined Use of MRI, fMRIand Cognitive Data for Alzheimer’s Disease: Preliminary Results. Appl. Sci..

