# Supplementary material for: Deep Learning-Based Post-Processing of Real-Time MRI to Assess and Quantify Dynamic Wrist Movement in Health and Disease
Source: Diagnostics (Basel). 2021 Jun 11;11(6):1077. doi: 10.3390/diagnostics11061077 (PMC8231139; doi:10.3390/diagnostics11061077)
Supplement: Supplementary file 1 [file diagnostics-11-01077-s001.zip › diagnostics-1253084-supplementary.pdf]

Supplementary Material

Title: Deep Learning-Based Post-Processing of Real-Time MRI to Assess and Quantify Dynamic Wrist Movement in Health and Disease

Authors: Karl Ludger Radke, Lena Marie Wollschläger, Sven Nebelung, Daniel Benjamin Abrar, Christoph Schleich, Matthias Boschheidgen, Miriam Frenken, Justus Schock, Dirk Klee, Jens Frahm, Gerald Antoch, Simon Thelen, Hans-Jörg Wittsack and Anja Müller-Lutz

Table S1. Overview of a selection of fast MR sequences with a short description of the basic principle.

**MR imaging technique**	**Method**
GeneRalized Autocalibrating Partial Parallel Acquisition (**GRAPPA**)	Sub-sampled k-space data are acquired for each coil element, then the signals of the different coil elements are summed together to form a complete k-space. After the missing k-space lines are recovered, the Fourier transform is applied to obtain the final image.
SENSitivity Encoding (**SENSE**)	Sub-sampled k-space data are acquired for each coil element, and an image with aliasing is determined for each coil by applying the Fourier transform. Then the different images are processed by matrix multiplications and a final image without aliasing is determined.
**k-t GRAPPA** and **k-t SENSE**	Analogous method to GRAPPA and SENSE, in addition to the spatial correlations, temporal correlations are used to speed up the measurement even more.
volumetric interpolated breath-hold (**VIBE**)	Gradient echo sequence that allows accelerated data acquisition with destruction of the transverse signal (by adding spoiler gradients along the slice and readout directions) and data interpolation.
true fast imaging with steady state precession (**TrueFISP**)	Sequence in which balanced gradients are used along all spatial directions so that the gradient-induced net dephasing over a TR interval is zero. Thus, by choosing a TR that is significantly shorter than T1 and T2, the sequence readout occurs in steady state.
highly under-sampled **FLASH** (fast low-angle shot)	Gradient echo sequence that uses small excitation angles as well as a short TR time to achieve a constant longitudinal equilibrium magnetization that is maintained for an arbitrarily long measurement time. This allows sequential single acquisitions. Using nonlinear inverse reconstruction with temporal regularization, it is possible to perform autocalibration of coil sensitivity as well as to use highly undersampled k-spaces data for image reconstruction.

Table S2. Summary of linear mixed model results for the fixed effects of gender, hand side, hand angle, height, and age on SL and LT gap widths of healthy volunteers during active radioulnar motion.

	**SL gap width [mm]**			**LT gap width [mm]**		
**Fixed effects**	**Estimate**	**Standard error**	**p-value**	**Estimate**	**Standard error**	**p-value**
Constant term	0.813	0.960	0.398	-2.058	0.83	0.014
Gender: male	-0.097	0.114	0.398	-0.145	0.098	0.141
Gender: female		redundant parameter			redundant parameter	
Hand Site: left	-0.624	0.063	**<0.001**	0.034	0.054	0.526
Hand Site: right		redundant parameter			redundant parameter	
angle	-0.002	0.001	**0.002**	-0.003	0.001	**<0.001**
height	0.006	0.006	0.296	0.019	0.005	**<0.001**
age	0.002	0.003	0.45	0.006	0.003	0.025

Video S1. Continuous alternating active radioulnar abductions (“waving”) of the left wrist of a healthy volunteer as imaged and processed by the framework. The sequence parameters for the real-time sequence were: repetition time: 4.5 ms, echo time: 2.5 ms, flip angle: 7°, 21 spokes.
